# Understanding the Mid-Infrared Spectra of Protic Ionic Liquids by Density Functional Theory

**DOI:** 10.1021/acs.jpcb.4c05084

**Published:** 2024-11-14

**Authors:** Yingzhen Chen, Christian Rodenbücher, Adrien Morice, Fabian Tipp, Piotr M. Kowalski, Carsten Korte

**Affiliations:** † Institute of Energy Technologies—Electrochemical Process Engineering (IET-4), Forschungszentrum Jülich GmbH, Jülich 52425, Germany; ‡ RWTH Aachen University, Aachen 52062, Germany; § Institute of Energy Technologies—Theory and Computation of Energy Materials (IET-3), Forschungszentrum Jülich GmbH, Jülich 52425, Germany; ∥ Jülich Aachen Research Alliance, JARA Energy & Center for Simulation and Data Science (CSD), Jülich 52425, Germany

## Abstract

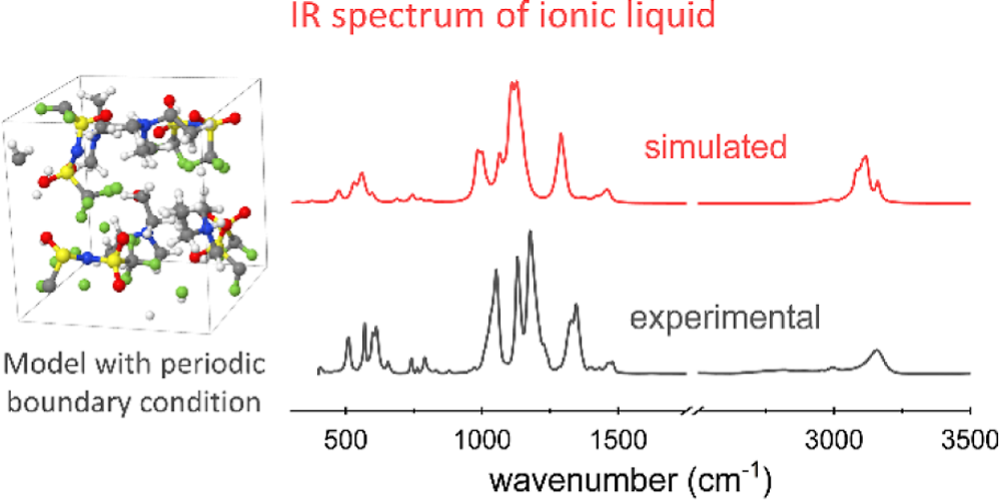

Protic ionic liquids (PILs) are promising candidates as electrolytes for proton exchange polymer membrane fuel cells. In order to optimize their properties, a detailed understanding of the molecular interactions within the bulk and at the electrode–electrolyte interface is needed, which can be obtained by infrared spectra. A prerequisite for extracting information on the molecular structure and inter- or intramolecular interactions from an experimental spectrum is a reasonable interpretation of the observed spectral features. Here, we employed density functional theory to understand the vibration modes of PILs composed of ammonium cations and different counteranions. Different from the previous calculation methods performed on small cluster model systems consisting of isolated species, a periodically repeated system of four ion pairs was used in order to approximate the bulk liquid environment. The computed frequencies and IR intensity match well with the corresponding experimental spectra, allowing for its proper interpretation, especially the characteristic features of the interionic interaction. The presented approach enables accurate computation of a variety of ionic liquid systems in a highly efficient way.

## 1 Introduction

Ionic liquids have attracted broad research interests due to their widespread applications, *e.g.*, as polar solvents in organic synthesis or as a lubricant with negligible vapor pressures.^1−3^ Recently, an increasing number of studies are focusing on the use of ionic liquids as electrolytes for electrochemical energy conversion and storage,^4,5^ due to their high electrochemical stability, high thermal stability, and nonflammability. Among different types of ionic liquids, protic ionic liquids (PILs), consisting of a cation with a Brønsted-acidic proton, have been considered as potential electrolytes for high-temperature proton exchange membrane fuel cells.^6−8^ The properties and bulk structures of ionic liquids are complex due to varieties of intermolecular interactions such as Coulomb forces, hydrogen bonds, van der Waal forces, and steric repulsion. These interactions lead to the formation of ion pairs and nanoscaled domains due to ordering phenomena of cations and anions in the 10 nm range.^9^ The related features can be revealed by means of spectroscopic techniques, such as infrared spectroscopy (IR).^10,11^ However, the interpretation of the experimental vibrational spectra of ionic liquids is challenging. In this context, atomistic simulations based on density functional theory (DFT) have been employed on a regular basis to assist the interpretation of IR spectra.^11−13^

To assign the vibrational peaks observed in the experimental IR spectra of ionic liquids, DFT calculations on single isolated cation and anion species are often performed and the results are used as reference data.^10,14−17^ This approach can identify some features of the measured spectrum resulting from the basic vibrational modes of the ion but could not contribute to simulating the spectral signatures arising from the interaction of anions and cations in a condensed phase. DFT calculations performed on ions pair have also been widely applied.^18−23^ Although those improved the description of IR spectrum, the vibrational frequencies and IR intensities computed by this method often cannot reproduce the measured spectra sufficiently.^20^ A more realistic approach to consider the interaction in a liquid phase consists of simulating the ionic species using a polarizable continuum model.^24^ In such an approach, the ion pair (cluster) is placed in a void that is embedded in a dielectric environment with the experimental permittivity constant of the bulk liquid. The dielectric interaction of the ionic charges of the simulated ion pair with the other ions in the surrounding bulk is modeled as an averaged interaction with a continuous polarizable medium.^24^ Owing to the lack of the exact value of the dielectric constants of the PILs, the dielectric constants of polar solvents (*e.g.*, dimethylformamide, ε_r_ = 37.22) and nonpolar solvents (*e.g.*, tetrahydrofuran, ε_r_ = 7.43) were applied in such calculations.^25^ Applying an inaccurate dielectric constant may result in an incorrect prediction of the positions and number of absorption peaks. In particular, for PILs, the possible proton transfer in PILs leads to hydrogen bond networks. This is the case for PIL based on alkylammonium cations, in which strong hydrogen bonds exist between the active proton of the cation and the anion. The N–H stretching is sensitive to the environment and interionic interaction with the neighboring ions, resulting in various distinguishable local dielectric environments.

In order to overcome the limitations of the aforementioned methods, we applied an approach based on DFT simulations of a system consisting of four ion pairs with periodic boundary conditions so as to obtain a better approximation of a bulk-like liquid environment. A series of diethylmethylammonium [Dema]^+^-based PILs with anions of different basicities and charge densities, *i.e.*, bis(trifluoromethanesulfonyl) imide [TFSI]^−^ (bistriflimide), trifluoromethanesulfonate [TfO]^−^ (triflate), hydrogen sulfate [HSO_4_]^−^, and methanesulfonate [MsO]^−^ (mesylate), have been studied. This enabled a quantitative analysis of the mid-IR spectra as well as of the interionic interactions.

## 2 Methods

### 2.1 Computational Methods

The IR spectra of four ionic liquids with the same cation but different counteranions were simulated with density functional perturbation theory (DFPT) using the Quantum ESPRESSO code with a plane-wave basis set.^26^ The Perdew–Burke–Ernzerhof (PBE) exchange–correlation functional was applied.^27^ The plane–wave cutoff energy was set to 30 Ry and the core electrons were replaced with ultrasoft pseudopotentials.^28^ To model four ion pairs, the initial configurations were obtained by performing classical molecular dynamics (MD) simulation using the LAMMPS software package.^29^ A unit cell with periodic boundaries in an isothermal–isobaric (*NPT*) ensemble was used for the preliminary relaxation. The temperature was set to be 298.15 K and the initial edge size of the cubic simulation box was 10 Å. The interactions between the ionic liquid molecules were modeled with the Optimized Potential for Liquid Simulations-All Atom (OPLS-AA) force field,^30−35^ and the parameters are listed in the Supporting Information. An equilibration run was performed in 300,000 steps with 1 fs time steps in *NPT* conditions, using a Nosé–Hoover thermostat. As depicted in Table 1, the calculated densities of PILs after equilibration were in good agreement with experimental data found in the literature (within 6%),^36−38^ which reflects the reliability of the applied force field.

**Table 1:** Comparison of Calculated Densities of PILs with the Experimental Ones^a^

PIL	ρ_20 °C_, literature (g cm^–3^)	ρ_MD_, this work
[Dema][TfO]	1.291^36^	1.344 g cm^–3^ (+4.1%)
[Dema][MsO]	1.135^36^	1.116 g cm^–3^ (−1.7%)
[Dema][HSO_4_]	1.235^37^	1.304 g cm^–3^ (−5.6%)
[Dema][TFSI]	1.453^37^	1.507 g cm^–3^ (+3.7%)

a Adapted from reference.^36,37^ Copyright [2014] [2016], American Chemical Society.

The obtained atomic configurations were taken as input structures for the DFT simulations and a structural relaxation has been performed using the Quantum ESPRESSO code.^26^ The calculations were performed by using the same periodic boundary conditions as those for the classical MD simulations. The relaxation of the atomic positions converged to a value for the residual forces of 1 × 10^–4^ Ry Bohr^–1^ for each atom. The vibrational analysis was performed on the DFT-optimized structures (see Figure S1) using DFPT as implemented in the PHonon tool of Quantum ESPRESSO package. In order to compare with the experimental spectra, the calculated, discrete IR spectra (based on a finite set of vibrational frequencies) were convoluted with the Lorentz profile curves with 20 cm^–1^ full width at half-maximum (fwhm). As a reference for comparing the results, the spectra of an isolated single PIL cation and anion as well as a single ion pair were also calculated, respectively. In order to prevent any interaction between the neighboring images, the isolated molecular species were computed in a cubic supercell with a cell length of 40 Å.

### 2.2 Infrared Spectroscopy Measurement

An FTIR spectrometer (Thermo Fisher Scientific, USA) with an attenuated total reflection unit (Monolithic diamond GladiATR, PIKE technologies, USA) was employed to record the IR spectra of ionic liquids. The spectra were collected in the range between 400 and 4000 cm^–1^ with a spectral resolution of 4 cm^–1^. A total of 32 scans were averaged per spectrum.

## 3 Results and Discussion

**Figure 1:**
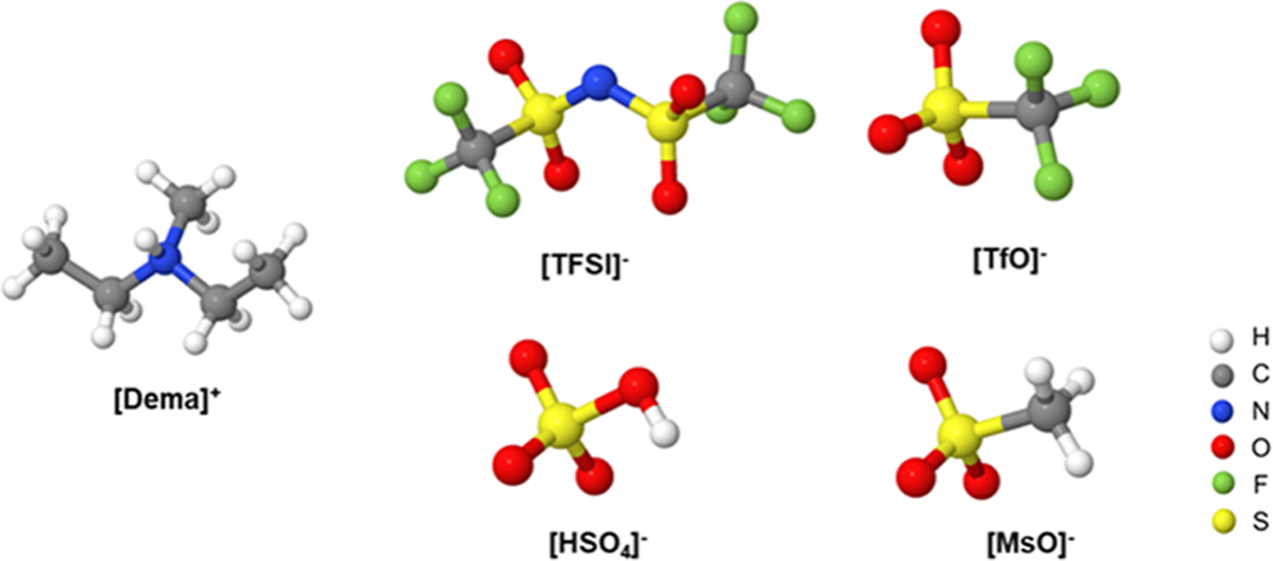
DFT-optimized structures of the [Dema]^+^ cation and [TFSI]^−^, [TfO]^−^, [HSO_4_]^−^, and [MsO]^−^ anion.

**Figure 2:**
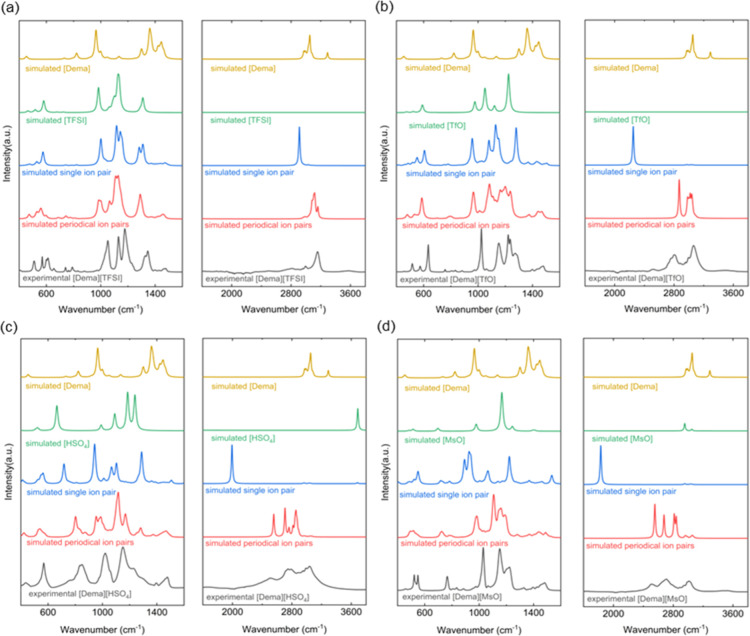
Simulated IR spectra of the PILs: (a) [Dema][TFSI], (b) [Dema][TfO], (c) [Dema][HSO_4_], and (d) [Dema][MsO]; yellow curve: simulation of one cation; green curve: simulation of one anion; blue curve: simulation of one single ion pair; red curve: simulation of four ion pairs with periodical boundary conditions; and black curve: experimental spectra.

Figure 1 illustrates the lowest-energy geometries of the [Dema]^+^ cation and of the different anions, *i.e.*, [TFSI]^−^, [TfO]^−^, [HSO_4_]^−^, and [MsO]^−^. The calculated IR spectra of the isolated cations and anions are plotted in Figure 2 as yellow curves and green curves, respectively. It is clear that the ν(NH) stretching vibration of an isolated single [Dema]^+^ cation is located at 3291 cm^–1^. The asymmetric and symmetric stretching vibrations of the C–H bonds of the methyl and ethyl groups, *i.e.*, the combined modes of ν_as_(CH_3_), ν_s_(CH_3_), ν_as_(CH_2_), and ν_s_(CH_2_), overlap in the range of 2900–3100 cm^–1^.^1^ The overlapping results in broad bands centered at 3055 and 2990 cm^–1^. In the low wavenumber region, the peak at 1361 cm^–1^ is caused by the δ(N–H) bending mode, whereas the broad band at 1445 cm^–1^ is contributed by various bending modes of C–H, *i.e.*, combined modes of δ(CH_3_), τ(CH_3_), and ω(CH_3_) from the methyl group and δ(CH_2_), τ(CH_2_), and ω(CH_2_) from the ethyl groups. The bands at 965 and 822 cm^–1^ are primarily attributed to the combined ν_as_(C–N–C) asymmetric stretching mode and ν(C–C) stretching modes.

As shown in the calculated spectrum of an isolated [TFSI]^−^ anion, three intense peaks are found in the region of 800–1400 cm^–1^. The absorption peak at 1309 cm^–1^ corresponds to the δ_as_(SO_2_) asymmetric stretching mode. The broad peak at 1129 cm^–1^, accompanied by a shoulder at 1101 cm^–1^, results from an overlap of various C–F and S–O stretching modes, *e.g.*, ν_s_(CF_3_), ν_as_(CF_3_), and ν_s_(SO_2_). The peak at 983 cm^–1^ corresponds to the ν_as_(S–N–S) asymmetric stretching mode. A medium peak is also found at 581 cm^–1^, which can be assigned to the ν_s_(S–N–S) symmetric stretching mode and δ(SO_2_) bending. The correspondence of the outlined spectral features calculated for the isolated ions to the experimental spectra is not obvious.

The simulated spectrum of isolated single pair of PIL is shown as a blue curve in Figure 2. The anion-type-dependent shifts of the [Dema]^+^ cation can be observed. The peak positions of these bands decrease in the order with [Dema][TFSI] > [Dema][TfO] > [Dema][HSO_4_] > [Dema][MsO], which correlates with increasing basicity of their counteranions. The frequency shifts can be related to different interaction strengths caused by different anions, which was also seen in previous studies of far-IR spectra of similar compounds.^41−47^ Although simulations of single ion pairs lead to an improvement over results obtained with the isolated ions, the computed peak positions do not match well the position of the broad peaks between 2000 and 3600 cm^–1^ observed in the experimental spectra.

The spectra computed for periodical repeated systems of four ion pairs to approximate a bulk environment are plotted as red curves in Figure 2. In the calculated spectrum of [Dema][TFSI], intense bands can be observed at 1290, 1123, 999, and 559 cm^–1^, see Figure 2a. The shapes of these bands are similar, and their peak positions are almost the same with a shift of less than 25 cm^–1^, as found for the calculated spectrum of a single isolated [TFSI]^−^ anion. This indicates that the fingerprint signals in the spectral region of 800–1400 cm^–1^ are primarily contributed by the [TFSI]^−^ anions. The ν(N–H) stretching mode from the cation can be found in the region of 3000–3200 cm^–1^, overlapping with the C–H stretching modes. Thus, there is a red shift of about 200 cm^–1^ compared to the frequency of the ν(N–H) stretching mode of a single isolated [Dema]^+^ cation. On the other hand, good match of the frequencies computed with the quasiharmonic approximation to the observed frequencies indicates suppression of anharmonic effects in a condensed phase. The δ(N–H) bending mode is blue-shifted and shows up at 1345 cm^–1^. The superposition of ν_as_(C–N–C) asymmetric stretching modes and ν(C–H) stretching modes results in the appearance of one broad but weak absorption peak centered at 747 cm^–1^. The calculated spectrum, based on four ion pairs and periodic boundary conditions, agrees better with the important features of the experimental spectrum than when a comparison is made with the spectra calculated for isolated ions and ion pairs. The details of the comparison of the experimental spectrum and the calculated one are presented in Table 2.

**Table 2:** Wavenumber of the IR Band Observed in the Experimental Spectra Compared to the DFT-Calculated Wavenumber Calculation with the Periodic Boundary Condition^a^

Exp. wavenumber (cm^–1^)		Cal. wavenumber (cm^–1^)	vibration modes of [Dema][TFSI]	Exp. wavenumber (cm^–1^)		Cal. wavenumber (cm^–1^)	vibration modes of [Dema][TfO]
3157	m	3117	ν(NH)	3062	s	3039	ν(NH)
2993	w	2983	ν(CH)	2994	m	2983	ν(CH)
1479	w	1345	δ(NH)	2801	s	2862	ν(NH)
1346	s	1290	ν_as_(SO_2_)	1479	m	1469	δ(NH)
1177	s	1123	ν_as_(CF_3_), ν_as_(SO_2_)	1274	m	1236	ν_as_(SO_3_)
1131	s	1066	ν_as_(CF_3_)	1221	s	1164	ν_s_(CF_3_)
1052	s	999	ν_as_(SNS)	1153	s	1083	ν_as_(CF_3_)
791	w	747	δ(CH), ν(CNC)	1024	s	966	ν_s_(SO_3_)
741	w	688	ν_s_(SNS)	757	w	788	δ(CH), ν(CNC)
612	m	559	δ_ip_(SO_2_)	634	s	587	ν(CS)
570	m	531	δ_op_(SO_2_)	574	m	534	δ(SO_3_)
510	m	474	δ(CF_3_)	516	m	481	δ(CF_3_)

Exp. wavenumber (cm^–1^)		Cal. wavenumber (cm^–1^)	vibration modes of [Dema][HSO_4_]	Exp. wavenumber (cm^–1^)		Cal. wavenumber (cm^–1^)	vibration modes of [Dema][MsO]
3036	s	2855	ν(OH), ν(CH)	3005	s	2962	ν(CH)
2987	m	2760	ν(NH)	2708	s	2676	ν(NH)
2745	s	2707	ν(NH)	2507	s	2553	ν(NH)
2501	m	2555	ν(OH)	1479	s	1488	δ(NH)
1477	m	1468	δ(NH)	1210	m	1189	ν_as_(CNC), δ(CH)
1225	m	1169	ν_as_(CNC), δ(CH)	1151	s	1107	ν_as_(SO_3_)
1150	s	1116	ν_as_(SO_3_)	1030	s	982	ν_s_(SO_3_)
1021	s	988	ν_s_(SO_3_)	765	m	724	ν(CS)
849	s	802	δ(SOH)	550	s	517	ω(SO_3_)
568	s	540	δ(SO_3_)	523	s	495	τ(SO_3_)

a The symbol s, m, and w indicate the strong, medium, and weak peak observed in the spectra, respectively, and the notation ν_s_, ν_as_, δ_ip_, δ_op_, ω, and τ represent the symmetric stretching, asymmetric stretching, in-plane bending, out–of plane bending, wagging, and twisting modes, respectively.

The IR band profiles calculated for the other [Dema]-based ionic liquids, [Dema][TfO], [Dema][HSO_4_], and [Dema][MsO], also match well the experimental spectra, especially in the wavenumber range of 400–1600 cm^–1^, see Figure 2b–d. The assignments of the peaks are also listed in Table 2. The intense peaks observed at 1274, 1221, 1153, 1024, and 634 cm^–1^ in the experimental spectrum of [Dema][TfO] can be assigned to vibration modes of [TfO]^−^, *i.e.*, the asymmetric stretching ν_as_(SO_3_), symmetric stretching ν_s_(CF_3_), asymmetric stretching ν_as_(CF_3_), symmetric stretching ν_s_(SO_3_), and wagging mode ω(SO_3_), respectively. In the experimental spectrum of [Dema][HSO_4_], the intense peaks at 1150, 1021, 849, and 568 cm^–1^ can be assigned to the asymmetric stretching ν_as_(SO_3_), the symmetric stretching ν_s_(SO_3_), and the bending mode δ(SOH). In the spectrum of [Dema][MsO], the intense peaks at 1151 and 1030 cm^–1^ can be assigned to the asymmetric stretching ν_as_(SO_3_) and symmetric stretching ν_s_(SO_3_), whereas the peak at 765 cm^–1^ corresponds to stretching ν(CS). This indicates again that the strong bands in the low wavenumber region are primarily contributed by the vibration modes from the corresponding anion of the ionic liquid.

According to the calculations, the C–N–C stretching and C–H bending modes of the cation can be found in the low-frequency region. The intensity is lower compared to the peaks assigned to the anions and are only visible as weak peaks in the range of 700–800 cm^–1^ in the spectra of [Dema][TFSI] and [Dema][TfO]; in other wavenumber ranges, these peaks are hidden due to an overlap with the peaks of the anion vibration modes. On the other hand, the C–H stretching modes can be found in the high wavenumber region. However, they have a lower intensity with respect to the N–H stretching modes and partially overlap with these. For example, in the experimental spectra of [Dema][TfO], the peaks related to the C–H stretching modes are found at 2994 and 2954 cm^–1^ and are superposed by a broad peak from the stretching mode of N–H. The assignment of these C–H stretching modes is in agreement with experimental studies performed on a deuterium substitution. In the IR spectrum of deuterated [Dema][TfO], *i.e.*, with a (C_2_H_5_)_2_CH_3_N-D^+^ cation, the relatively weak signals of the C–H stretching mode can be clearly observed, as the N-D signal is shifted to lower wavenumbers compared to N–H.^40^

The N–H bending mode of the [Dema]^+^ cation gives rise to a medium peak at about 1480 cm^–1^ for the investigated PILs. The frequency of the N–H stretching modes varies for different ion pairs, *i.e.*, for different anions. As shown by the DFT simulations based on four ion pairs, each cation has a different frequency for the N–H stretching mode, giving rise to four different peaks in the spectrum. The N–H stretching modes can be found at 3161, 3124, 3118, and 3106 cm^–1^ in the case of [Dema][TFSI], whereas in the case of [Dema][TfO], these peaks are shifted to lower frequencies, 3038, 3017, 3000, and 2981 cm^–1^. For [Dema][HSO_4_] and [Dema][MsO], they appear at 2891, 2855, 2844, and 2814 cm^–1^ and at 2840, 2813, 2676, and 2553 cm^–1^, respectively. In comparison with the experimental spectra, broad bands in the corresponding regions are measured. In the experimental spectrum of [Dema][TFSI], a broad band exists centered at 3157 cm^–1^. Two broad bands at 3062 and 2801 cm^–1^ were observed for [Dema][TfO], but at 3036 and 2745 cm^–1^ for [Dema][HSO_4_] and at 3005 and 2708 cm^–1^ for [Dema][MsO]. The computed peak positions match well the experimentally measured features, which indicates that variable structural arrangements in the direct vicinity of the cations of the ionic liquid are an origin of the variability of the high-frequency, H–N vibrational mode.

Overall, the simulated results based on a model with four ion pairs in a periodic box describe well the experimental vibrational spectra of all investigated PILs and represent a significant improvement compared with the calculations based on isolated ions or a single ion pair. This modeling approach is able to approximate the environment of the ions in the bulk of an ionic liquid and reveals their characteristic features due to the interionic interaction between the cations and anions. All of the ionic liquids considered are composed of a [Dema]^+^ cation. The results indicate that the N–H bond in the [Dema]^+^ cation is highly influenced by the presence of the counteranion and its structure. The shift of the ν(N–H) stretching mode is found in the experimental and calculated IR spectra. In the case of an isolated [Dema]^+^ cation, the N–H stretching mode is located at 3290 cm^–1^. The presence of an anion in close proximity leads to the formation of a hydrogen bond with the oxygen atoms N–H···O of the anion. The presence of the H-bond is shown by X-ray diffraction and NMR spectroscopy. The effect of H-bond on intermolecular interaction was also observed by Fumino et al. in the range from 100 to 300 cm^–1^ in the far-IR spectra of PILs based on [(CH_3_)_3_NH]^+^ cation.^45−47^ The formation of a hydrogen bond induces a general weakening of the N–H bond strength accompanied by an increase in the N–H bond length. Accordingly, the N–H stretching shifts to a lower frequency, *i.e.*, it is red-shifted. Regarding the extent of the observed red shift, there is a clear dependence on the basicity of the counteranion. A higher basicity of the anion decreases the charge density of the cation and strengthens the hydrogen bond. The broad N–H bands observed in the high-frequency region are explained by a superposition of slightly different N–H vibration modes in local perturbed environments due to thermal fluctuations in the real liquid. This is because the simulation using four ion pairs and periodic boundary conditions yields peak positions of the N–H stretching signals that correspond well with the position of the broad band observed in the experimental spectrum. Obviously, the model system consisting of four ion pairs has its limitations and cannot cover the entire range of N–H vibration modes realized in the real bulk environment of an ionic liquid, and for this reason, we cannot expect a perfect reproduction of observed high frequency, N–H mode feature.

## 4 Conclusions

We employed DFT with a periodically repeated system of four ion pairs to determine the vibration modes of bulk PILs. The simulated frequencies and intensities match well with the experimental spectra and represent a significant improvement when compared with simulations based only on isolated ions or small isolated clusters. The results indicate that the intense peaks in the low frequency region (below 1400 cm^–1^) are mainly contributed by the vibration modes of the corresponding anion. There are only weak IR peaks due to the vibrational modes of the cation. The medium peak found at around 1480 cm^–1^ in all investigated ionic liquids can be assigned to the N–H bending mode. The N–H stretching frequencies give rise to peaks in the spectral range of 2500–3500 cm^–1^. The shift in the N–H stretching in the ammonium-based ionic liquids was found to be dependent on the strength of the interaction between the cation and anion by hydrogen bonds. The formation of hydrogen bonds with the anion induces a weakening of the N–H strength and so leads to a shift toward a lower wavenumber in the spectra. The calculations reveal that the thermal fluctuations in the local environment of the hydrogen bond lead to a distribution of slightly modified bond strengths and, thus, to a broad IR band. This study demonstrates that it is possible to accurately simulate the structural features of a bulk ionic liquid and its IR spectral response with a model system of a few ions in a periodically repeated simulation box.

## Supporting Information Available

Force field parameters for MD simulation and DFT-optimized structure of ionic liquids (PDF)

## Footnotes

1 Symbols used to specify a vibration mode: symmetric stretching (ν_s_), asymmetric stretching (ν_as_), symmetric bending (δ_s_), asymmetric bending (δ_as_), wagging (ω), and twisting (τ).^39^
