# Supplementary material for: Understanding the Mid-Infrared Spectra of Protic Ionic Liquids by Density Functional Theory
Source: J Phys Chem B. 2024 Nov 14;128(47):11723–9. doi: 10.1021/acs.jpcb.4c05084 (PMC11613440; doi:10.1021/acs.jpcb.4c05084)
Supplement: Supplementary file 1 — jp4c05084_si_001.pdf [file jp4c05084_si_001.pdf]

Supplementary Information for

# Understanding the Mid-Infrared Spectra of Protic Ionic Liquids by Density Functional Theory

Yingzhen Chen,[a,b] Christian Rodenbücher, [a] Adrien Morice, [a] Fabian Tipp[c,d], Piotr M. Kowalski,[c,d] and Carsten Korte*[a,b]

[a] Institute of Energy Technologies – Electrochemical Process Engineering (IET-4), Forschungszentrum Jülich GmbH, 52425 Jülich, Germany

[b] RWTH Aachen University, 52062 Aachen, Germany

[c] Institute of Energy Technologies – Theory and Computation of Energy Materials (IET-3), Forschungszentrum Jülich GmbH, 52425 Jülich, Germany.

[d] Jülich Aachen Research Alliance, JARA Energy & Center for Simulation and Data Science (CSD), 52425 Jülich, Germany

## Contents

Table S1: Force Field parameters for [Dema][1] ..................................................... S2
Table S2: Force Field parameters for [TfO][2] ..................................................... S3
Table S3: Force Field parameters for [TFSI][2] ..................................................... S4
Table S4: Force Field parameters for [MsO][3] ..................................................... S5
Table S5: Force Field parameters for [$HSO_4$][4–6] ..................................................... S6
Figure S1: DFT-optimized structure of four ion pairs of the PILs ..................... S7

The models of ionic liquids for molecular dynamics (MD) simulation were based on Optimized Potential for Liquid Simulations- All Atom (OPLS-AA) force field. The potential energy is a sum of the intramolecular part (harmonic bond stretching and angle bending terms, dihedral interactions terms) and the intermolecular part for the non-bonded interactions (Lennard-Jones terms for van der Waals interactions and Coulomb terms for electrostatic interactions).

## Table S1: Force Field parameters for [Dema][1]

| Non-Bonded parameters | | | |
|---|---|---|---|
| **Atom** | **q (e)** | **σ (Å)** | **ε (kcal.mol$^{-1}$)** |
| $H_a$ | +0.31 | 0.00 | 0.00 |
| $H_b$ | +0.13 | 2.50 | 0.0300 |
| $H_c$ | +0.06 | 2.50 | 0.0300 |
| N | +0.03 | 3.250 | 0.1700 |
| $C_a$ | -0.17 | 3.50 | 0.0660 |
| $C_b$ | -0.05 | 3.50 | 0.0660 |

| Bond stretching parameters | | |
|---|---|---|
| **Bond** | **$K_b$ (kcal.mol$^{-1}$.Å$^{-2}$)** | **$R_0$ (Å)** |
| H-N | 434.00 | 1.0100 |
| C-N | 367.00 | 1.4710 |
| C-H | 340.00 | 1.0900 |
| C-C | 268.00 | 1.5290 |

| Angle-bending parameters | | |
|---|---|---|
| **Angle** | **$K_a$ (kcal.mol$^{-1}$.rad$^{-2}$)** | **θ (deg)** |
| C-N-H | 35.00 | 109.50 |
| C-C-H | 37.50 | 110.70 |
| H-C-H | 33.00 | 107.80 |
| C-C-N | 80.00 | 111.20 |
| C-N-C | 50.00 | 113.00 |
| H-C-N | 35.00 | 109.50 |

| Dihedral angle parameters | | | |
|---|---|---|---|
| **Dihedral angle** | **$V_1$ (kcal.mol$^{-1}$)** | **$V_2$ (kcal.mol$^{-1}$)** | **$V_3$ (kcal.mol$^{-1}$)** |
| H-C-N-H | 0.00 | 0.00 | 0.261 |
| H-C-C-N | 0.00 | 0.00 | 0.384 |
| C-C-N-H | 0.00 | 0.00 | 0.347 |
| C-C-N-C | 1.740 | -0.157 | 0.279 |
| H-C-N-C | 0.00 | 0.00 | 0.366 |
| H-C-C-H | 0.00 | 0.00 | 0.318 |

Note: $H_a$: hydrogen bonded to N. $H_b$: hydrogen bonded to $C_a$. $H_c$: hydrogen bonded to $C_b$. $C_a$: carbon bonded to N. $C_b$: carbon bonded to $C_a$.

## Table S2: Force Field parameters for [TfO][2]

| Non-Bonded parameters | | | |
|---|---|---|---|
| **Atom** | **q (e)** | **σ (Å)** | **ε (kcal.mol$^{-1}$)** |
| C | +0.2692 | 3.50 | 0.0660 |
| F | -0.1637 | 2.95 | 0.0530 |
| S | +1.1887 | 3.55 | 0.2500 |
| O | -0.6556 | 2.96 | 0.2100 |

| Bond stretching parameters | | |
|---|---|---|
| **Bond** | **$K_b$ (kcal.mol$^{-1}$.Å$^{-2}$)** | **$R_0$ (Å)** |
| C-F | 441.92 | 1.323 |
| C-S | 233.03 | 1.818 |
| S-O | 637.07 | 1.437 |

| Angle-bending parameters | | |
|---|---|---|
| **Angle** | **$K_a$ (kcal.mol$^{-1}$.rad$^{-2}$)** | **θ (deg)** |
| F-C-F | 93.33 | 107.1 |
| S-C-F | 82.93 | 117.1 |
| C-S-O | 103.97 | 102.6 |
| O-S-O | 115.80 | 118.5 |

| Dihedral angle parameters | | | |
|---|---|---|---|
| **Dihedral angle** | **$V_1$ (kcal.mol-1)** | **$V_2$ (kcal.mol-1)** | **$V_3$ (kcal.mol-1)** |
| | 0.00 | 0.00 | 0.3468 |

## Table S3: Force Field parameters for [TFSI][2]

| Non-Bonded parameters | | | |
|---|---|---|---|
| **Atom** | **q (e)** | **σ (Å)** | **ε (kcal.mol$^{-1}$)** |
| N | -0.66 | 3.25 | 0.169886 |
| O | -0.53 | 2.96 | 0.20986 |
| C | +0.35 | 3.50 | 0.06595493 |
| F | -0.16 | 2.95 | 0.0529641 |
| S | +1.02 | 3.55 | 0.249833 |

| Bond stretching parameters | | |
|---|---|---|
| **Bond** | **$K_b$ (kcal.mol$^{-1}$.Å$^{-2}$)** | **$R_0$ (Å)** |
| C-F | 441.506831 | 1.323 |
| C-S | 235.26331 | 1.818 |
| S-O | 636.644013 | 1.437 |
| N-S | 371.763799 | 1.570 |

| Angle-bending parameters | | |
|---|---|---|
| **Angle** | **$K_a$ (kcal.mol$^{-1}$.rad$^{-2}$)** | **θ (deg)** |
| F-C-F | 93.269363 | 107.1 |
| S-C-F | 82.879562 | 111.8 |
| C-S-O | 103.89801 | 102.6 |
| O-S-O | 115.720887 | 118.5 |
| O-S-N | 94.224747 | 113.6 |
| C-S-N | 97.449168 | 100.2 |
| S-N-S | 80.132833 | 125.6 |

| Dihedral angle parameters | | | |
|---|---|---|---|
| **Dihedral angle** | **$V_1$ (kcal.mol-1)** | **$V_2$ (kcal.mol-1)** | **$V_3$ (kcal.mol-1)** |
| F-C-S-O | 0.00 | 0.00 | 0.346566 |
| S-N-S-O | 0.00 | 0.00 | -0.00358269 |
| F-C-S-N | 0.00 | 0.00 | 0.315754 |
| S-N-S-C | 7.8277 | -2.488775 | -0.7631130 |

## Table S4: Force Field parameters for [MsO][3]

| Non-Bonded parameters | | | |
|---|---|---|---|
| **Atom** | **q (e)** | **σ (Å)** | **ε ($kcal.mol^{-1}$)** |
| C | -0.140 | 3.50 | 0.03296075 |
| H | 0.000 | 2.50 | 0.0150473 |
| S | 1.180 | 3.55 | 0.249833 |
| O | -0.680 | 3.15 | 0.099957 |

| Bond stretching parameters | | |
|---|---|---|
| **Bond** | **$K_b$ ($kcal.mol^{-1}.Å^{-2}$)** | **$R_0$ (Å)** |
| C-H | 40.60382 | 1.09 |
| C-S | 235.26331 | 1.792 |
| S-O | 636.644013 | 1.455 |

| Angle-bending parameters | | |
|---|---|---|
| **Angle** | **$K_a$ ($kcal.mol^{-1}.rad^{-2}$)** | **θ (deg)** |
| H-C-H | 39.40959 | 107.8 |
| H-C-S | 46.610797 | 107.3 |
| C-S-O | 103.89801 | 104.5 |
| O-S-O | 115.720887 | 114.0 |

| Dihedral angle parameters | | | |
|---|---|---|---|
| **Dihedral angle** | **$V_1$ (kcal.mol-1)** | **$V_2$ (kcal.mol-1)** | **$V_3$ (kcal.mol-1)** |
| H-C-S-O | 0.00 | 0.00 | 0.388125 |

# Table S5: Force Field parameters for $[HSO_4]$[4–6]

| Non-Bonded parameters | | | |
|---|---|---|---|
| **Atom** | **q (e)** | **σ (Å)** | **ε (kcal.mol$^{-1}$)** |
| H | +0.404 | 3.195 | 0.0000 |
| $O_a$ | -0.651 | 3.4046 | 0.1700 |
| $O_b$ | -0.541 | 3.4046 | 0.1700 |
| S | +1.045 | 4.03 | 0.3440 |

| Bond stretching parameters | | |
|---|---|---|
| **Bond** | **$K_b$ (kcal.mol$^{-1}$.Å$^{-2}$)** | **$R_0$ (Å)** |
| $O_b$-H | 553.0000 | 0.988 |
| $O_a$-S | 700.0000 | 1.499 |
| $O_b$-S | 450.0000 | 1.6925 |

| Angle-bending parameters | | |
|---|---|---|
| **Angle** | **$K_a$ (kcal.mol$^{-1}$.rad$^{-2}$)** | **θ (deg)** |
| $O_a$-S-$O_a$ | 104.000 | 115.2 |
| S-O-H | 74.0000 | 109 |
| $O_a$-S-$O_b$ | 74.0000 | 105.933 |

| Dihedral angle parameters | | | |
|---|---|---|---|
| **Dihedral angle** | **$V_1$ (kcal.mol-1)** | **$V_2$ (kcal.mol-1)** | **$V_3$ (kcal.mol-1)** |
| O-S-O-H | 0.00 | 0.00 | 0.7500 |

Note: The parameters are generated from LigParGen web server.[6] $O_a$: oxygen bonded to the sulfur. $O_b$: oxygen bonded to both sulfur and hydrogen

**Figure S1: DFT-optimized structure of four ion pairs of the PILs**

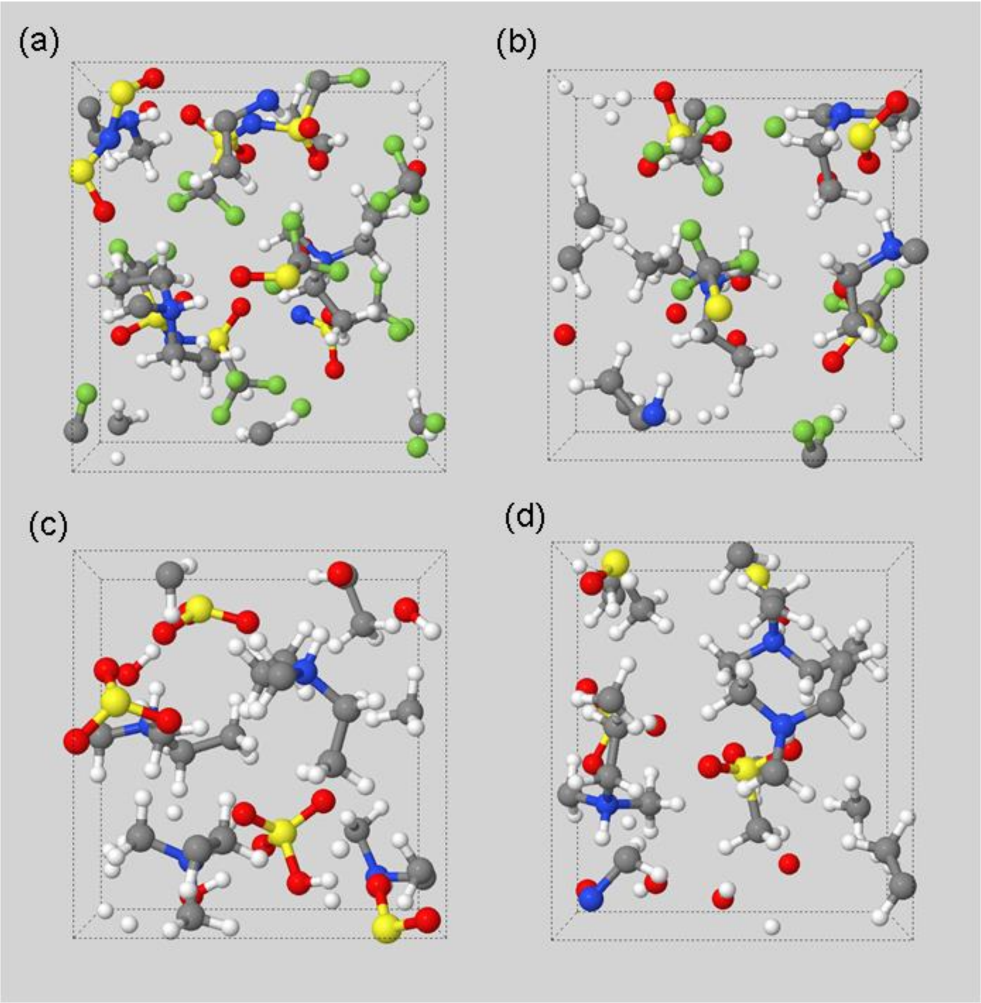


Figure S1 Illustrations of DFT-optimized structure of four ion pairs: (a) [Dema][TFSI]; (b) [Dema][TfO]; (c) [Dema][$HSO_4$]; (d) [Dema][MsO].

# References


(1) Nasrabadi, A. T., Gelb, L. D. Structural and Transport Properties of Tertiary Ammonium Triflate Ionic Liquids: A Molecular Dynamics Study. *J. Phys. Chem. B* **2017**, *121*, 1908–1921.

(2) Canongia Lopes, J. N., Pádua, A. A. H. Molecular Force Field for Ionic Liquids Composed of Triflate or Bistriflylimide Anions. *J. Phys. Chem. B* **2004**, *108*, 16893–16898.

(3) Lopes, J. N. C., Padua, A. A. H., Shimizu, K. Molecular force field for ionic liquids IV: trialkylimidazolium and alkoxycarbonyl-imidazolium cations; alkylsulfonate and alkylsulfate anions. *J. Phys. Chem. B* **2008**, *112*, 5039–5046.

(4) Jorgensen, W. L., Tirado-Rives, J. Potential energy functions for atomic-level simulations of water and organic and biomolecular systems. *Proc. Natl. Acad. Sci. U.S.A.* **2005**, *102*, 6665–6670.

(5) Dodda, L. S., Vilseck, J. Z., Tirado-Rives, J., Jorgensen, W. L. 1.14*CM1A-LBCC: Localized Bond-Charge Corrected CM1A Charges for Condensed-Phase Simulations. *J. Phys. Chem. B* **2017**, *121*, 3864–3870.

(6) Dodda, L. S., Cabeza de Vaca, I., Tirado-Rives, J., Jorgensen, W. L. LigParGen web server: an automatic OPLS-AA parameter generator for organic ligands. *Nucleic Acids Res.* **2017**, *45*, W331-W336.
